# Metal(loid)s in tap-water from schools in central Bangladesh (Mirpur): Source apportionment, water quality, and health risks appraisals

**DOI:** 10.1016/j.heliyon.2023.e15747

**Published:** 2023-04-28

**Authors:** Md. Joynal Abedin, Rahat Khan, Md. Abu Bakar Siddique, Abdul Hadi Al Nafi Khan, Md. Tariqul Islam, Md. Bazlar Rashid

**Affiliations:** a Centre for Higher Studies and Research, Bangladesh University of Professionals (BUP), Mirpur Cantonment, Mirpur, Dhaka 1216, Bangladesh; b Institute of Nuclear Science and Technology, Bangladesh Atomic Energy Commission, Savar, Dhaka 1349, Bangladesh; c Institute of National Analytical Research and Service (INARS), Bangladesh Council of Scientific and Industrial Research (BCSIR), Dhanmondi, Dhaka 1205, Bangladesh; d Geological Survey of Bangladesh, Segunbaghicha, Dhaka 1000, Bangladesh

**Keywords:** Tap water from educational institutes, Metal(loid)s load, Tap water quality, Source apportionment, Supply pipe-line scaling, Health risks on exposure

## Abstract

Considering the health risks originating from the exposure of metal(loid)s in tap-water and the concomitant vulnerability of school-going students, 25 composite tap water samples from different schools and colleges of central Bangladesh (Mirpur, Dhaka) were analyzed by atomic absorption spectroscopic technique. Elemental abundances of Na, Mg, K, Ca, Cr, Mn, Fe, Co, Ni, Zn, As, Cd, and Pb in the studied tap water samples varied from 4520 to 62250, 2760–29580, 210–3000, 15780–78130, 1.54–5.32, 7.00–196, 2.00–450, 0.04–1.45, 8.23–24.4, 0.10–813, 0.10–10.5, 0.002–0.212, and 1.55–15.8 μgL^−1^, respectively. Dissolved metal(loid)s' concentrations were mostly within the national and international threshold values with few exceptions which were also consistent with the entropy-based water quality assessment. Multivariate statistical approaches demonstrated that hydro-geochemical processes like water-rock interactions mostly govern the major elemental (Na, Mg, K, Ca) compositions in tap water. However, anthropogenic processes typically control the trace elemental compositions where supply pipeline scaling was identified as the major source. Cluster analysis on sampling sites separated two groups of schools and colleges depending on their establishment years where tap water from older schools and colleges possesses relatively higher levels of metal(loid)s. Hence, gradual pipeline scaling on a temporal scale augmented the metal(loid)s' concentrations in tap-water. In terms of non-carcinogenic health risks estimation, studied tap-water seems to be safe, whereas elemental abundances of Pb and As can cause carcinogenic risks to school-going people. However, progressive deterioration of water quality by pipeline scaling will be supposed to cause significant health risks in the future, for which preventative measures should be adopted.

## Highlights

• Dissolved metal(loid)s in tap water from educational institutions were measured.
• Major elemental abundances were mostly governed by the water-rock interactions.
• Supply pipe-line scaling control the dissolved trace metal(loid)s in tap water.
• Pb and As in tap water can cause carcinogenic health risks on continuous exposures.
• Younger age group are more vulnerable toward the dissolved metal(loid)s than adults.

## Graphical abstract

**Figure:**
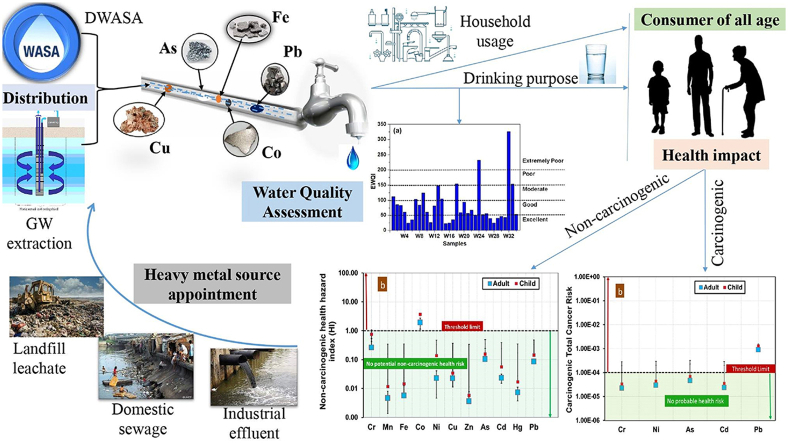

## 1 Introduction

For the sustenance of human life on planet earth, the safety of water is the prime concern. Billions of people’s physical and environmental health are threatening in both developing and developed countries due to the rise of water pollution [1]. About 3.4 million people die worldwide each year from waterborne diseases [2]. In this regard, UNICEF has proclaimed the Sustainable Development Goal (SDG) [3], where SDG-6 seeks to confirm the access to clean and safe water & sanitation for all focusing on their sustainable managements by the year 2030 [4]. Currently, the worldwide COVID-19 pandemic has further demonstrated the great importance of sanitation and adequate access to clean and safe water for diseases prevention. However, the uncontrolled existence of metal(loid)s (e.g., Cr, Fe, Mn, Co, Ni, Zn, Cd, As, Pb) in water is making it harder to achieve the SDG-6 [3].

Considering the persistence, non-carcinogenic, and carcinogenic physiological impacts, dissolved trace metal(loid)s in drinking and domestic water are of great concern [[5], [6], [7]]. Previous studies [[8], [9], [10]] demonstrated that a high intake of Pb can generate immutable intellectual impairment in young children and infants, even at low concentration (below 10 mg/dL) of Pb in blood. Moreover, the neurotoxic effects of Pb are most noticeable in young children, pregnant women, and fetuses [10]. The poisoning due to Pb was also regarded as a classic disease where mainly the central nervous system and the gastrointestinal tract-related symptoms were commonly seen in children and adults [11]. Other than Pb, As in water can cause the disease called ‘arsenicosis’, whereas high consumption of Mn and Cu through drinking water for a long time may create neurological disorders [12], e.g., Alzheimer’s [13] while Cd can damage the skeletal system (Itai-Itai disease) [14] and cause mental and physiological growth retardation among children in chronic exposure, whereas Ni can affect the pulmonary system and cause dermal problems as well. Nevertheless, health hazards from Cr cannot be overlooked as it can cause diseases such as lung and gastrointestinal cancers [15]. On the contrary, Co metal exists in vitamin-B12 which is essential for human’s physiological growth [16,17], however excess consumption of vitamin-B12 and thus Co may induce several pathological problems such as the overproduction of red blood cells, abnormal thyroid artery, and coronary diseases [18]. Similarly, consumption of excessive amount of Fe and Zn could lead to ‘hemochromatosis’ [19] and dermal problems [14], respectively. Most noticeably, for almost all health-related risks originating from water, the younger age group is more vulnerable than the older people [6,7]. Water-borne diseases cause much-unexpected death, particularly among children, which is widespread in Bangladesh [20]; every year more than one hundred thousand children die due to water-related diseases in Bangladesh [21]. In this consideration, evaluation of potentially toxic elemental abundances in the tap water of educational institutions is indispensable to understand the water pollution status, sources, and to adopt the potential remediation approaches.

Several studies [5,[22], [23], [24], [25], [26], [27]] were carried out on tap water in different countries like Egypt, France, Iran, Pakistan, Poland, and Bangladesh which demonstrated health-risk due to potentially toxic metal(loid)s in tap-water. But to be very specific, targeting the younger age group people, evaluation of metal(loid)s in tap-water with associated health risks are very scarce. Few studies had been carried out in primary schools of southern Bangladesh to assess the drinking water quality and found that the school-going children were vulnerable to As, Fe, Mn, and Zn [[28], [29], [30]].

Dhaka is a highly populated (23,234 people/km^2^) [[31], [32], [33]] megacity of Bangladesh, and about 95% population of Dhaka depends on tap water from various sources for their daily household work and other essential purposes [34]. Mirpur is one of the densely populated parts of this megacity where the number of educational institutions is also very high. The water sources of this area are adversely affected by rapid urbanization, industrialization, uncontrolled sewage release, and agricultural activities [35]. However, considering the higher vulnerability of younger age people, evaluation of tap water quality of educational institutions and associated health risks on toxic metal(loid)s exposures have not hitherto been demonstrated in this densely populated portion of the megacity Dhaka. Hence, the key objectives of this work are (1) to quantify the concentration of potentially toxic metal(loid)s in tap-water of educational institutions, (2) to assess the quality of tap water, (3) to appraise the potential origins, and the distributions of the measured metal(loid)s, and (4) to evaluate the preliminary health risks emphasizing on the school-going children owing to the exposures of the toxic metal(loid)s from the tap-water. The outcome of the present work will create a current pollution scenario with affiliated health risks which will help the policymakers to formulate an outline plan for supplying safe water to educational institutions.

## 2 Experimental

### 2.1 Study area

**Fig. 1:**
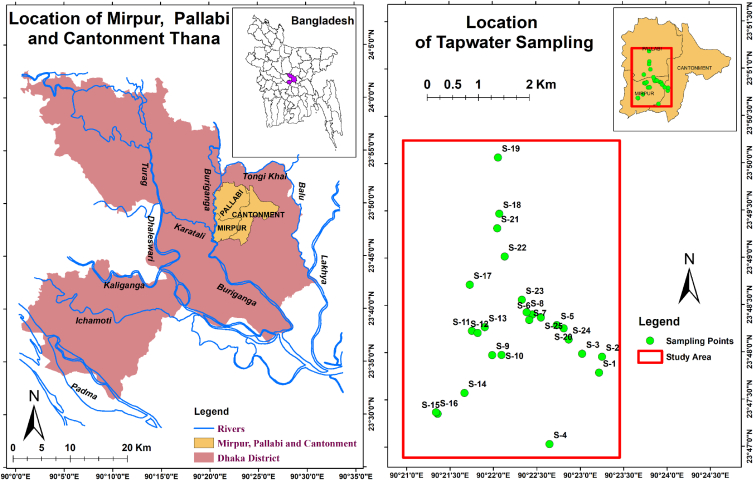
Sampling locations (educational institutions) in and around Mirpur, Dhaka, Bangladesh.

The area of study is situated on the northeast side of the megacity Dhaka, which is placed in the central zone of Bangladesh (Fig. 1). The study area falls mainly under the supply zone IV and X of Dhaka Water Supply & Sewerage Authority (DWASA), where groundwater serves the large portion of the demand and only small part covered from surface water. There is a single surface water treatment plant (Saidabad water treatment plant) from where part of the study area receives water supply. This plant receives water from heavily polluted Lakhya (/Shitalakhya) river via artificial canal. The treatment begins with aerobic biological pre-treatment to remove ammonia. At the later phases, pre-chlorination is done before coagulation and flocculation by aluminium sulphate and lime, respectively. Particle, colloids and planktons removed through sedimentation, flocculation and sludge blankets in pre-treatment clarification units. Water from clarification unit passed through sand filters, and chlorine and lime added to disinfect and control pH at the final phase of treatment [36]. However, zone IV and X withdraw groundwater through more than 100 deep tube wells inside Dhaka city, and the study area gets water supply from these two zones. Water from these deep tube wells is supplied directly to households after chlorination at the withdrawal site (at the face of pumps) [36]. In recent days, groundwater is based Tetuljhora-Bhakurta project satisfies a part of the water demand for Mirpur, Pallabi and Cantonment area. In addition to chlorination plant, Tetuljhora-Bhakurta project includes iron removal facilities as well [37].

The sampling took place mostly in the Mirpur Thana area. However, a significant part of Pallabi Thana and a small part of Cantonment Thana were covered in the process. The overall studied region has been presented through a rectangular block, which extends from 90,^0^20′57.25″ E to 90,^0^23′25″ E longitude and 23,^0^46′52.5″ N to 23,^0^50′15″ N latitude. The total area of the rectangular portion covers approximately 26.2 km^2^ of Dhaka city, which is seemingly one-third of the total area under the three Thanas (75.4 km^2^). The total population of these three Thana is approximately 1.25 million according to the last national survey in 2011 [38]. A hot and humid tropical weather is prevailing in Mirpur, Dhaka and it belongs to the tropical Savanna climate. Four discrete seasons are noticed in the studied area as in other parts of the country [6]: pre-monsoon, monsoon, post-monsoon, and dry season. Long-term climate data (1981–2010) indicate that annual temperature varies from 13.1 to 33.8 °C in the Dhaka district. Annual mean precipitation is 2085 mm, while 65% of yearly precipitation takes place during the monsoon [39]. Dhaka city is relatively flat land where the topographic elevation varies in the range of 0.5–12 m PWD (Public Work Datum) [40]. The study area is surrounded by the Buriganga river at the west, Balu river at the east, Lakhya (/Shitalakhya) at the south, and Tongi Khal at the north [40]. These rivers, wetlands, and direct rainfall used to play as vital sources for groundwater recharge in this area, i.e., Dhaka district. However, loss of wetlands and expansion of urban settlement with the increase in impermeable land cover have squeezed the scope of subsurface recharge [40,41]. Moreover, with progressive industrialization and swift urbanization by a high influx of population, water demand has increased manifold. Hence, subsequent lowering of the groundwater table has resulted in this area. The depletion scenario is highly critical in the Mirpur area where the drawdown of the water table has been observed as 42.2 m from 1995 to 2018 [42]. Other than recharge issues, the surface water quality has been highly affected by unplanned sewage systems, water-logging, untreated industrial waste discharge, lack of management of domestic waste, etc., which are accustomed scenarios of this area for quite a long-time [35,[43], [44], [45]]. The surface water quality may impose an adverse influence on the groundwater quality through surface water influx into the groundwater. DWASA is the key authority for the supply of tap water in the studied region. However, some educational and commercial institutions may have private deep tube well facilities. DWASA mainly relies on groundwater (70% of the total supply) and the distribution takes place through the overhead tanks in different parts of the study area. Surface water is used in the monsoon period while achievement of the desired quality of drinking water cannot be possible since heavy contamination from heavy metals and organic materials heavily degraded the quality of water in the source areas [46]. Groundwater quality in the Mirpur area is also at risk in recent years, since the indication of mixing of pollutants carried by surface water has been reported in the area [36].

### 2.2 Sample collection, pre-treatments, and measurements

A total of 25 composite samples of tap water were collected randomly from 25 different educational institutions (schools and colleges) located in the studied area (Fig. 1) during dry season. Sample information along with their ancillary data given in Table S1. Water samples were collected directly from taps of each of the randomly selected educational institutions in 2L pre-cleaned high-quality polyethylene bottles. The sample bottles were soaked overnight with 10% HNO_3_ solution, washed carefully with deionized water, and dried in the air. Before collecting each tap water sample, the bottle was rinsed properly with the corresponding tap water of the individual educational institution. To make the samples as representative as possible, the sample bottles were filled up with the 3–5 separate aliquots of the corresponding water samples collected at a few minute intervals while keeping the flowing water tap open, and the bottles were immediately capped tightly [47]. All possible precautions were taken to avoid unwanted contamination during the collection of tap water samples. After collecting all samples, concentrated nitric acid was mixed with the tap water samples (2 mL/L) to prevent absorption toward the wall of the sample containers. Collected samples were then transported to the analytical laboratory on the same day and stored at 4 °C until the analysis of chemical elements.

The elemental concentrations of Na, Mg, K, Ca, Cr, Fe, Mn, Co, Zn, As, Ni, Cd, and Pb in tap-water sample were measured using Atomic Absorption Spectrophotometer (AAS) and for the analysis, the samples were prepared following the works published earlier [48,49]. In AAS, all elements were measured employing the absorption technique while Na and K were measured by the emission technique. For the determination of Na, Mg, K, and Ca, the samples were diluted while determining the other element, the samples were pre-concentrated to estimate the concentrations of the elements within the range of their calibration curves in the measurements. The concentrations of elements such as Na, K, Mg, Mn, Ca, Fe, and Zn in tap-water samples were measured using the flame technique of AAS (Model: AA240FS, Varian, Australia) whereas the elements, Cr, Co, Ni, Cd, and Pb were analyzed by AAS equipped with an electrical heating system in graphite furnace (Model: GTA120-AA240Z, Varian, Australia). Concentration of As in tap-water samples was measured using the hydride vapor generation technique of AAS (Model: SpectrAA 220 equipped with ETC-60 and VGA-77, Varian, Australia). While estimating the concentration of chemical elements by the aforementioned techniques, the quality control schemes were applied following the same procedures as those in the previous works [6,[49], [50], [51], [52], [53]]. The spike recovery of the measured elements was in the range of 94–104%. Other analytical parameters such as condition of element-specific hollow cathode lamp, limit of quantification, limit of detection, calibration range, and measurement uncertainty for all the analyzed chemical elements are provided in the Supplementary Table S2.

### 2.3 Water quality apprising index

The concept of entropy is expressed by Ref. [54] as an important criterion for determining the information/uncertainties that can envisage the result of probabilistic existence [55]. The entropy principle involves the bellow mentioned steps to elaborate the water quality [56,57]. For appraising the entropy-weight for m (i = 1, 2, …, m) number of water samples with n (j = 1, 2, …, n) number of the measured parameter, eigenvalue matrix, X can be adorned using Eq. (1).

(1) $$X=\left[\begin{array}{ccc} x_{11} & x_{12}\cdots & x_{1n}\\ x_{21} & x_{22}\cdots & x_{2n}\\ \begin{array}{c} \vdots \\ x_{m1} \end{array} & \begin{array}{c} \vdots \ddots \\ x_{m2}\ldots \end{array} & \begin{array}{c} \vdots \\ x_{mn} \end{array} \end{array}\right]$$

To minimize the influences of different units and concentrations of the measured parameters, a normalization approach of efficiency type [58] (Eq. (2)) was attained to convert the eigenvalue matrix (X) into a single standard grade matrix (Y) (Eq. (3)).

(2) $$Y_{ij}=\frac{X_{ij}-{\left(X_{ij}\right)}_{\min}}{{\left(X_{ij}\right)}_{\max}-{\left(X_{ij}\right)}_{\min}}$$

(3) $$Y=\left[\begin{array}{ccc} y_{11} & y_{12}\cdots & y_{1n}\\ y_{21} & {xy}_{22}\cdots & y_{2n}\\ \begin{array}{c} \vdots \\ y_{m1} \end{array} & \begin{array}{c} \vdots \ddots \\ y_{m2}\ldots \end{array} & \begin{array}{c} \vdots \\ y_{mn} \end{array} \end{array}\right]$$

Hereafter, the ratio of the measured parameter index (P_ij_), information entropy (e_j_), and entropy weight (ω_j_) can be computed by Eqs. (4)–(6).

(4) $$P_{ij}=\frac{Y_{ij}}{\sum_{i=1}^{m}Y_{ij}}$$

(5) $$e_{j}=-\frac{1}{\ln\left(m\right)}\sum_{i=1}^{m}\left(P_{ij}{\times lnP}_{ij}\right)$$

(6) $$\omega_{j}=\frac{1-e_{j}}{\sum_{j=1}^{m}\left(1-e_{j}\right)}$$

The scale of quality ranking for the measured parameters (j) can be computed from the analyzed data (C_j_) and the standard data (S_j_) by Eq. (7).

(7) $$q_{j}=\frac{C_{j}}{S_{j}}\times 100$$

From Eqs. (6) and (7), EWQI can be computed by Eq. (8).

(8) $$EWQI=\sum_{j=1}^{n}\omega_{j}q_{j}$$

Based on the value of EWQI, tap water samples can be categorized into five classes: (1) EWQI < 50 is excellent to use for drinking purposes; (2) 50 ≤ EWQI < 100 is good and suitable for drinking; (3) 100 ≤EWQI <150 is moderate and suitable for domestic, irrigation, and industrial uses; (4) 150 ≤EWQI <200 is assigned as poor and not suitable for drinking; and (5) EWQI ≥ 200 is considered as extremely poor-quality water and prohibited for human consumptions [6,42,59].

### 2.4 Health risk estimating indices

One significant step in the assessment of human health-risk is the assessment of the pollutants exposures to humans which facilitates the entrance of toxic pollutants to the human body through several exposure pathways such as ingestion, inhalation, and dermal or skin contact [51,60,61], and thus potentially affecting the human health [26]. The health risk computation is generally depending on the determination of risk level and is usually revealed by the non-carcinogenic and carcinogenic health-risks [62]. The assessment of health risks induced by the metal(loid)s dissolved in the tap-water is generally considered due to the oral ingestion directly and through dermal absorption by exposure [63]. Following Ref. [64], the exposure doses due to the direct ingestion (ADD_ingestion_) and dermal absorption (ADD_dermal_) can be represented by Eqs. (9), (10), respectively.

(9) $${ADD}_{ingestion}=\frac{C_{w}\times IR\times {Abs}_{g}\times EF\times ED}{BW\times AT}$$

(10) $${ADD}_{dermal}=\frac{C_{w}\times SA\times K_{p}\times EF\times ET\times ED\times 10^{-3}}{BW\times AT}$$

where, C_w_, IR, EF, SA, ET, ED, BW, AT, Abs_g_, and K_p_ represent the trace elements concentrations (μg L^−1^), ingestion rate (L day^−1^), exposure frequency (days year^−1^), area of exposed skin (cm^2^), exposure time (h day^−1^), exposure duration (in years), body weight (in kg), average time for non-carcinogens (days), gastrointestinal absorption factor, and dermal permeability coefficient (cm h^−1^), respectively. Individual values of each aforementioned parameter are summarized in Table S3. In addition, non-carcinogenic health hazards can be evaluated from the calculation of Hazard Quotient (HQ) for both oral ingestion (HQ_ingestion_) & dermal exposure (HQ_dermal_) using the following Eqs. (11), (12), (13).

(11) $${HQ}_{ingestion}=\frac{{ADD}_{ingestion}}{{R_{f}D}_{ingestion}}$$

(12) $${HQ}_{dermal}=\frac{{ADD}_{dermal}}{{R_{f}D}_{dermal}}$$

(13) R_f_ D_dermal_ = R_f_ D_ingestion_ × Abs_g_

Here, R_f_D_ingestion_ & R_f_D_dermal_ are the reference doses for individual chemical elements (μg kg^−1^ day^−1^) and are also tabulated in Table S3 [64,65]. Nevertheless, total potential non-carcinogenic health hazard can be estimated by the Hazard Index (HI) using Eq. (14).

(14) $$HI=\sum_{i-1}^{n}\left({HQ}_{ingestion}+{HQ}_{dermal}\right)$$

For HI value of greater than 1, there is a probable hazardous risk of the contaminated water. Hence, the non-carcinogenic health hazards can only be considered when the HQ and HI values are greater than 1. Further, the additive and/or interactive health effects from multiple chemical elements can be derived by adding the value of the Total Hazard Quotient (THQ) for specific metal; hence, the Total Target Hazard Quotient (TTHQ: [66]) can be estimated by Eq. (15):

(15) TTHQ = THQ_Cr_ + THQ_Mn_ + THQ_Fe_ + THQ_Co_ + THQ_Ni_ + THQ_Cu_ + THQ_Zn_ + THQ_As_ + THQ_Cd_ + THQ_Hg_ + THQ_Pb_

The TTHQ value of higher than 1 imposes the likelihood of inconvenient health consequences whereas the TTHQ value of less than 1 demonstrates no probable health hazard due to the exposure of the considered chemical elements at the current consumption rates [66,67]. Furthermore, Carcinogenic Risk (CR) from both oral ingestion and dermal exposure can be calculated using Eqs. (16), (17), respectively and the total carcinogenic risk can be obtained by Eq. (18).

(16) CR_ingestion_ = ADD_ingestion_ × CSF_ingestion_

(17) CR_dermal_ = ADD _dermal_ × CSF_dermal_

(18) CR_total_ = CR_ingestion_ + CR_dermal_

Here, CSF (mg/kg/day)^−1^ represents the Cancer Slope Factor. In this study, CR for both ingestion and dermal was measured only for As (CSF for ingestion: 0.0015; CSF for dermal: 0.00366) as the values were mentioned by Ref. [68]. However, the tolerable or acceptable range of carcinogenic risks is usually 1.0 × 10^−6^–1.0 × 10^−4^ [64,65]. In this study, only As is considered for carcinogenic risk assessment. Because Bengal basin is well known for it’s arsenic contamination where more than 25 million people drink water containing more than 50 μg/L of arsenic, and likely ∼25 million people drink water with 10–50 μg/L arsenic [69].

### 2.5 Statistical approaches

The determined data of measured metal(loid)s were assessed statistically by SPSS software (IBM-Corporation, Version-20, Armonk, NY, USA). To evaluate the significant factors, interrelationships, and probable sources of contamination of the analyzed tap-water, multivariate methods of statistics such as principal component, cluster, and correlation analyses were utilized in this work [49,[70], [71], [72]]. The principal component analysis (PCA) was performed with the Varimax rotation method to extract the principal components (PCs) from the estimated data of the measured metal(loid)s to evaluate data variations, probable contamination sources, and degree of contamination in tap-water samples of the study area [56,[72], [73], [74]]. The cluster analysis (CA) was employed with Ward’s method and the rescaled linkage distance as a measure of similarity to explore the resemblance among the analyzed metal(loid)s originated probably from different sources [75,76]. Generated dendrogram from the CA provides a visual inspection of the various clusters based on the proximity of the data of the analyzed parameters. Pearson’s correlation matrix was also carried out to find the significant relations among the measured metal(loid)s [77] in the tap water of the study area.

## 3 Results and discussion

### 3.1 Chemical characteristics of tap water samples

Concentrations of the measured major (Na, Mg, K, and Ca) and potentially toxic (Cr, Fe, Mn, Co, Zn, Ni, As, Cd, Pb) elements in the tap-water from the educational institutions are shown in Table 1, Table 2, with their detail descriptive statistics, recommended values, and comparable literature. Major elemental abundances (n = 25) of Na, Mg, K, and Ca vary from 4520 to 62250, 2760–29580, 210–3000, and 15,780–78130 μg L^-1^, respectively where the relative standard deviations (in %) range from 24.6 to 47.0%. Mean Na-concentrations (29,292 μgL^−1^) of this study is relatively higher than the literature data mentioned in Table 2, except for the drinking water sources from Rangpur, Bangladesh [78] and Nigeria [79]. In contrast, the average concentration of K (2239 μgL^−1^) is relatively lower compared to the literature data, except for Ghana [80] and Saudi Arabia [81]. Similarly, the average content of Ca (41,584 μgL^−1^) is higher than in Rangpur, Bangladesh [78], Ghana [80], and Saudi Arabia [81], but lower than in Jamalpur, Bangladesh [82], France [25], and Nigeria [79]. However, mean Mg-contents (16,034 μgL^−1^) are considerably higher compared to the reported literature cited in Table 2: Jamalpur, Bangladesh [82], Rangpur, Bangladesh [78], France [25], Ghana [80], Nigeria [79] and Saudi Arabia [81] which is indicating the comparatively higher hardness of the studied water samples [[83], [84], [85], [86]].

**Table 1:** Elemental abundances (in μg L^−1^) in tap water samples collected from educational institutions (Mirpur, Bangladesh) along with their entropy water quality index (EWQI), descriptive statistics, and a comparison with those of internationally recommended values and previous relevant literature data.

Sample	Na	Mg	K	Ca	Cr	Mn	Fe	Co	Ni	Zn	As	Cd	Pb	EWQI
S-1	27,370	12,970	2630	35,030	3.57	19	318	1.38	9.30	704	5.00	0.078	6.35	26.9
S-2	32,310	14,060	2190	38,300	4.09	19	293	1.45	10.6	54.3	0.55	0.064	15.8	22.5
S-3	28,910	17,650	2070	42,250	2.71	10	380	1.26	8.60	21.0	0.15	0.109	9.50	19.6
S-4	24,200	14,690	2200	33,400	2.09	12	324	0.09	8.23	20.6	0.45	0.015	10.8	18.9
S-5	15,020	13,150	1820	29,480	2.99	26	58	0.57	12.0	813	0.10	0.072	4.78	12.5
S-6	56,030	26,210	2780	58,550	3.17	7	2	0.64	10.8	69.5	0.50	0.029	3.18	8.10
S-7	25,090	13,350	2020	34,030	2.09	7	26	0.20	10.6	4.00	1.05	0.003	7.85	10.5
S-8	59,780	27,330	2810	61,680	4.60	27	209	0.58	13.8	13.2	10.5	0.022	4.06	30.4
S-9	25,020	12,970	2310	33,800	2.40	196	100	0.40	10.3	24.2	2.38	0.053	3.91	18.6
S-10	50,250	24,800	2640	56,180	2.59	20	93	0.83	10.7	34.8	0.73	0.002	6.00	12.5
S-11	4520	2760	210	15,780	3.39	8	8	1.08	10.1	4.10	0.25	0.045	6.88	7.14
S-12	21,600	13,380	1970	30,280	2.36	31	220	0.07	24.4	139	2.90	0.046	3.03	18.1
S-13	22,490	13,020	2030	30,050	5.32	26	98	1.31	11.3	0.10	1.53	0.212	3.54	11.7
S-14	28,000	17,150	2370	36,330	4.28	85	450	0.44	13.2	120	0.68	0.063	3.93	22.2
S-15	31,100	17,780	2110	43,830	4.56	37	235	1.26	13.7	2.30	0.98	0.059	1.55	13.8
S-16	34,710	18,820	2230	57,930	1.88	41	274	0.23	11.6	0.70	0.83	0.020	15.5	23.0
S-17	21,470	15,170	1720	32,600	2.77	57	110	0.60	15.5	31.0	0.60	0.073	11.3	16.6
S-18	22,930	14,030	2180	42,550	3.25	39	11	0.49	12.0	8.70	0.55	0.065	4.25	8.67
S-19	20,860	11,770	1970	41,930	3.32	21	18	0.87	12.6	378	0.48	0.114	3.43	9.35
S-20	25,450	13,950	2350	42,880	1.90	10	86	0.08	9.44	75.3	0.93	0.060	4.22	10.1
S-21	25,690	14,440	3000	46,130	1.54	66	317	0.20	9.34	11.1	0.73	0.032	3.06	16.4
S-22	22,890	17,780	2410	49,750	3.78	33	56	0.04	10.9	4.80	0.55	0.008	4.87	10.0
S-23	62,250	29,580	2760	78,130	3.49	9	29	0.26	8.65	3.80	0.73	0.115	2.65	8.98
S-24	21,110	11,870	2990	34,180	1.76	160	384	1.04	8.41	193	0.65	0.041	4.19	22.4
S-25	23,240	12,180	2210	34,550	2.67	38	175	0.39	11.5	6.90	0.60	0.058	6.94	14.2
Error (%)^a^	2–4%	2–3%	2–5%	2–3%	5–7%	4–6%	2–6%	8–10%	6–9%	2–12%	2–13%	10–15%	2–6%

**Table 2:** Descriptive statistics of elemental abundances and the EWQI of studied water samples along with the internationally recommended values and previous relevant literature data.

	Na	Mg	K	Ca	Cr	Mn	Fe	Co	Ni	Zn	As	Cd	Pb	EWQI
This work														
Mean (n = 25)	29,292	16,034	2239	41,584	3.06	40.2	171	0.63	11.5	109	1.38	0.058	6.06	15.7
SD (1σ)	13,764	5786	550	13,114	0.99	46.0	139	0.46	3.3	213	2.16	0.045	3.81	6.40
RSD (%)	47.0	36.1	24.6	31.5	32.4	114.6	81.5	72.8	28.3	194.5	157.4	77.5	62.9	40.4
Median	25,090	14,060	2210	38,300	2.99	26.0	110	0.57	10.8	21.0	0.68	0.058	4.25	14.2
Min	4520	2760	210	15,780	1.54	7.00	2.00	0.04	8.23	0.10	0.10	0.002	1.55	7.14
Max	62,250	29,580	3000	78,130	5.32	196	450	1.45	24.4	813	10.5	0.212	15.8	30.4
Recommended Limit														
BIS [87]		100		75,000	50	100	300			5000	50	10	50	
ECR [88]	200,000	30,000–35000	12,000	75,000	50	100	300–1000		100	5000	50	5	50	
EPA [89]	200,000				50	50	200		20		10	5	10	
EU [90]	200,000				50				20		10	5	10	
WHO [12]	200,000		>3,000,000	100,000–300000	50	400	300	100	70	5000	10	3	10	
Literature Data														
Drinking Water: Primary Schools, Khulna, Bangladesh [30]						190	2180			230	2.4	<2	<2	
Potable Water: Primary Schools, Magura, Bangladesh [28]						144	2148.03				15.9			
Drinking Water: Primary Schools, Satkhira, Bangladesh [29]							1544.9				26			
Ground Water: Dhaka, Bangladesh [35]					209					21.007				
Drinking Water: Jamalpur, Bangladesh [82]	17,710	13,250	2350	43,490	6.00	1075	363			20		8	16	
Drinking Water: Noakhali, Bangladesh [91]					0.81	192			1.24	23.2	328	<0.05	0.17	
Drinking Water: Rangpur, Bangladesh [78]	39,100	7546	6510	12,630		684	7726			33.3	8.8			
Drinking Water: Northern China [92]					17.4	58.22	67	7.28	13.05	46.76	15.16	0.02	0.45	
Drinking Water: Dalian, China [93]									1.93	139.5	1.08	0.03		
Supply Water: Egypt [22]						430	350					5.25	15.5	
Tap Water: Metrpolitan, France [25]	22,300	8200	2640	76,000	<0.5	<0.5	26	<0.5	2.5	810	0.5	<0.5	1.8	
Groundwater: Atonsu-Kumasi, Ghana [80]	12,740	60	1900	5400	<0.006	<0.002	47			0.01		<0.002	22	
Tube well Water: Gangetic Basin, India [94]					8.60	199	2786	1.38		542	9	0.28	15	
Tap Water: Khorramabad, Iran [24]					5.08				3.47	47.01		0.43	3.2	
Drinking Water: Myanmar [95]						343	775			100			10	
Drinking Water: Nigeria [79]	50770.8	9585.6	7332.4	44,160	329		162.8			136			91.48	
Tap Water: Islamabad, Pakistan [27]							399		1	222	1.95	2.7	64.5	
Drinking Water: Northern Pakistan [16]					21.0	5.24		0.94	4.7	651.5		1.01	2.54	
Supply Water: Poland [23]					2.70							0.1	<1	
Groundwater: Middle Russia [96]					8.00		130					0.2		
Drinking Water: Saudi Arabia [81]	10,840	1570	740	16,260	18.0					89		61		
Groundwater: Vietnam [97]							3300				2			
EWQI Parameters														
Standard Data (S_j_)	200,000	35,000	12,000	300,000	50	400	300	100	70	5000	10	3	10	
Information Entropy (e_j_)	0.955	0.969	0.983	0.960	0.931	0.793	0.886	0.900	0.884	0.645	0.764	0.908	0.905	
Entropy Weight (ω_j_)	0.030	0.021	0.011	0.027	0.046	0.136	0.075	0.066	0.076	0.234	0.156	0.061	0.062	

Error (^a^Analytical uncertainty, in %).

Furthermore, the concentrations of the potentially toxic elements such as Cr, Mn, Fe, Co, Ni, Zn, As, Cd, and Pb vary from 1.54 to 5.32, 7.00–196, 2.00–450, 0.04–1.45, 8.23–24.4, 0.10–813, 0.10–10.5, 0.002–0.212, and 1.55–15.8 μgL^−1^, respectively with a long-range (28.3–157.4%) of relative standard deviations (in %). Mean concentrations of Cr (3.06 μgL^−1^), Mn (40.2 μgL^−1^), Fe (171 μgL^−1^), Co (0.63 μgL^−1^), As (1.38 μgL^−1^), and Cd (0.058 μgL^−1^) in the analyzed tap water were mostly lower compared to those of relevant literature data (Table 2). On the contrary, the average (n = 25) contents of Ni (11.5 μgL^−1^), Zn (109 μgL^−1^), and Pb (6.06 μgL^−1^) are relatively higher compared to those in most of the literature data.

### 3.2 Elemental distributions

The descriptive statistics regarding the concentrations of major ions and heavy metals have been presented in Table 2. Ca^2+^ (mean concentration: 41,584 μgL^−1^, n = 25) was found as the most abundant cation in the collected samples of tap water. The mean abundance (in μgL^−1^) of the major cations follows the order as Ca^2+^ > Na^+^ > Mg^2+^ > K^+^. The maximum concentrations of Na^+^ (62,250 μgL^−1^), Mg^2+^ (29,580 μgL^−1^), and Ca^2+^ (78,130 μgL^−1^) were found at S-23 (Shaheed Abu Taleb High School), whereas the maximum concentration of K^+^ (3000 μgL^−1^) was found at S-21 (Cosmo School). The central part of the study area and a part of the southwest show comparatively higher concentrations of the major cations except for K^+^, indicating more mineralization in those areas (Fig. S1). Other parts of the study area, especially, Pallabi and Cantonment areas show less abundance of the major cations. K^+^ distribution among the sampling sites is relatively more homogeneous than the other measured major elements, which is also concomitant with its lower RSD (24.6%, n = 25) (Fig. S1).

Fe (mean = 171 μgL^−1^) was the governing element among the trace metals, while significant presence from Zn (mean = 109 μgL^−1^) and Mn (mean = 40.2 μgL^−1^) was noticed in the tap water samples. The relative abundance for the analyzed trace elements obtains the order as Fe > Zn > Mn > Ni > Pb > Cr > Co > Cd. Maximum values of Fe (450 μgL^−1^), Zn (813 μgL^−1^), and Mn (196 μgL^−1^) were found in S-14 (Paikpara Govt. Primary School), S-5 (Banaful Adibasi Green Heart College), and S-1 (Monipur High Girls School and College), respectively. Although almost all the samples satisfy the WHO margin of As concentration in drinking water, S-8 (Senpara Porbota Govt. Primary School) shows a comparatively elevated concentration of As (10.5 μgL^−1^), which is slightly above the WHO recommended value. The spatial distribution (Arc-GIS: Inverse Distance Weighting interpolation technique) of the dissolved trace metal(loid)s indicates that Fe, Mn, Cr, Co, and Pb are predominantly partitioned in the southern portion of the studied sites, while Zn had a scattered concentration pattern (Fig. S2). The northern and western portions of the studied site possess a higher amount of Zn. However, As (RSD: 157.4%) and Cd (RSD: 77.5%) are mostly dominant in the central zone of the studied site. Noticeably, Ni possesses a relatively homogeneous distribution among the trace metal(loid)s (RSD: 28.3%). The southern part of the study area shows the presence of Fe and Pb in an elevated manner while following a similar distribution pattern at that part. Therefore, corrosion from galvanized iron pipes can be presumed in this part [98]. It is also logical to think about the potential intrusion from surface water pollutants [36] since significant levels of Fe and Pb have been observed in the surrounding surface water bodies [99,100]. Furthermore, the difference in the distribution pattern between Mn and Fe indicates that these two elements may not be of completely geogenic origin, while artificial sources may be responsible for their occurrence. The samples in the study area satisfy the WHO margin mostly. However, an improved water supply quality monitoring system should be initiated in the Mirpur region, since a contaminant plume has been suspected in prior studies [5,6,83] and an indication of potential intrusion of toxic elements has also been addressed in this study.

### 3.3 Water quality assessment

To draw a comprehensive water quality assessment, two different methods are adopted in this study: (1) comparing the analytical data of this study with those of corresponding national and international recommended values, and (2) information entropy-based water-quality assessment. In this investigation, the average concentrations (n = 25) of Na, K, Ca, Cr, Fe, Mn, Co, Zn, Ni, As, Cd, and Pb in tap-water samples were lower than the national & international threshold values presented in Table 2. Nevertheless, the analyzed average concentration value of Mg (16,043 μgL^−1^) was found significantly higher than the recommended limit of BIS [87] and lower than that of ECR [88]. In tap water sample S-23 (National Bangla High School), Ca concentration was found 78,130 μgL^−1^ which was higher than the threshold value of BIS [87] and ECR [88] but below the limit of WHO [12] (Table 2). In the case of Mn, the obtained values of samples S-14, S-17, and S-21 were found higher than the guideline value of EPA [89] while the obtained Mn-concentration of samples S-9 and S-24 were higher than those of the guideline values of BIS, ECR and EPA [[87], [88], [89]]. Fe concentrations in S-1, S-3, S-4, S-14, and S-24 were higher than the recommended values (300 μgL^−1^) of BIS and WHO [12,87] while sampling sites S-2, S-8, S-12, S-15, and S-16 possess higher contents of Fe compared to the recommended value (200 μgL^−1^) of EPA [89]. On the other hand, Ni contents in all the samples were lower than the lowest recommended limits (20 μgL^−1^) proposed by EPA [89] and EU [90], except for S-12 (24 μgL^−1^). Similarly, in all the tap-water samples, As content is significantly lower than the recommended limits (10 μgL^−1^) proposed by WHO, EPA, and EU [12,89,90], except for S-8 (10.5 μgL^−1^). However, sampling sites S-2, S-4, S-16, and S-17 possess a higher amount of Pb compared to the recommended value (10 μgL^−1^) of EPA, EU, and WHO [12,89,90].

Furthermore, to assess the quality of the collected tap-water samples, the EWQI was utilized since it is one of the most dependable and worldwide recognized methods as compared to the consensus-based weighing approach. Most importantly, EWQI can lessen the comparative error by recognizing the weight of every studied parameter logically and thus is considered as the more justified and acceptable techniques [6,58]. Two significant parameters related to EWQI *viz.*, information entropy (e_j_) and entropy weight (ω_j_) which are required to reveal the water quality in terms of EWQI are calculated and tabulated in Table 2. Moreover, the leading impactful measured parameter can be estimated by computing these two entropy parameters, such as, any parameter with higher ω_j_ and lower e_j_ value indicates that the parameter possesses heavier impact on the general water-quality [5]. Results of these two entropy parameters (Table 2) show that Zn, As, and Mn possess the higher ω_j-_value and lower e_j_ value which implies that these elements have the maximum impact on the overall water-quality relative to the other determined metal(loid)s. Based on the obtained values of ω_j_ and e_j_ (Table 2), the consequences of variables on overall water-quality follows the declining order as Zn > As > Mn > Ni > Fe > Co > Pb > Cd > Cr > Na > Ca > Mg > K. This is also expressed that major elements (Na, K, Mg, and Ca) have minimal impact on the quality of the tap water. Average values of EWQI for the analyzed tap water samples vary from 7.14 to 30.4, with an average of 15.7 ± 6.4 (±SD, n = 25), which indicated that currently considered educational institutions are obtaining water having ‘excellent’ quality for drinking.

### 3.4 Hydro-chemical evaluation and source apportionment of metal(loid)s in tap water

**Fig. 2:**
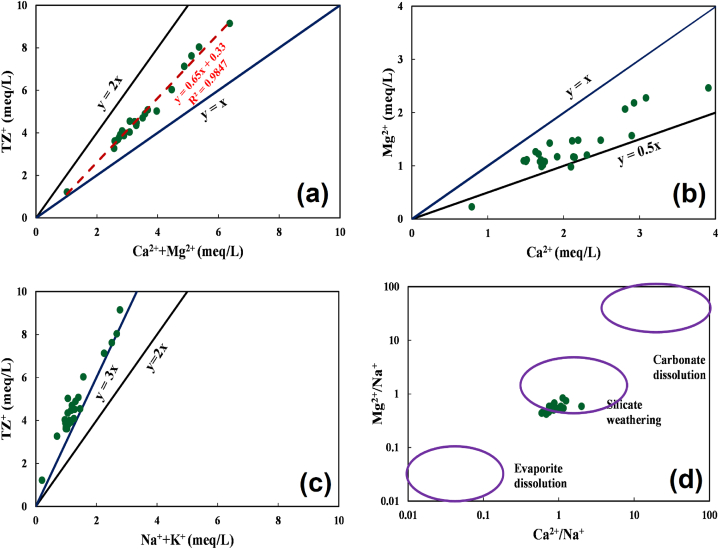
Bivariate plots of hydro-chemical parameter.

A set of bivariate plots have been utilized regarding the primary evolution of the water types in this study (Fig. 2). Bivariate plots using cations are often useful in understanding the water-rock interaction processes in the water. TZ^+^ (total cation) vs Ca^2+^ + Mg^2+^ plot in Fig. 2a shows that carbonate weathering is the major hydro-chemical process for the studied samples. The trend line equation reveals that the degree of mineralization in the groundwater was strongly induced by carbonate weathering as the regression coefficient value was near unity (R^2^ = 0.985). Moreover, the slope of the trend-line was found as 1.51, suggesting that nearly two-thirds of the cations have originated from the carbonate dissolution process [101]. Calcite weathering was the prevalent carbonate dissolution process as most of the samples were found in between y = x and y = 0.5x line in Fig. 2b [102]. Other than carbonate dissolution, the silicate weathering process is also involved in the source hydrochemistry of the supplied water in the study area, as evidenced from Fig. 2d showing the Na^+^-normalized plot of Mg^2+^ vs Ca^2+^ [103]. However, the share of silicate weathering is not that prominent since all the samples are placed above the y = 3x line (Fig. 2c), which indicates that silicate that the share of silicate weathering is mostly less than 33% in the hydro-chemical evolution of the water types [104].

For recognizing the potential sources of metal(loid)s dissolved in the analyzed tap-water, multivariate statistical approaches, e.g., principal component analysis (PCA), hierarchical cluster analysis (CA), and Pearson’s correlation matrices were utilized in this study. PCA functions as an extensive statistical tool in exploring the resemblance of data pattern for significant information regarding the particular study [105]. Hence, PCA was utilized to recognize the probable sources and clustering of the measured metal(loid)s in the collected samples of tap-water, which extracted five components from the analyzed data sets with Eigenvalues > 1 [106]. The reduction in the dimensions of initial data of the estimated chemical elements without considerable loss of original data information provided five loading factors and about 77.55% of the total variance is explained by those five factors (Table S4). PC1, which is accounted for 27.74% of the total variance, demonstrated strong positive loadings for Na, Mg, K, and Ca. About 80% of the tap-water in Dhaka city is supplied from the groundwater [107]. Thus, the component PC1 is assumed to represent the inherent hydro-geochemical characteristics of the water where water-rock interactions (e.g., dolomite weathering) govern the major cations in tap water [6]. Significant (p < 0.01) positive inter-element correlations among the major elements (Table S5) as well as Cluster-1 in hierarchical cluster analysis (Fig. S3a) also support the statistical and hydrogeological findings from PC1. However, other components (PC2-PC5) are cumulatively explaining 49.81% of the total variance which are indicating the differential anthropogenic incorporations of trace metal(loid)s in the analyzed tap water. Relative standard deviations (28.3–194.5%, n = 25) for the measured trace elements are significantly higher than the corresponding analytical uncertainties, which is indicating the significant spatial variations. It is also implying that different sampling points have faced diverse point or non-point sources of trace elements. PC2 explained 16.28% of the total variance demonstrating strong positive loadings for Cr, Co, and Cd, which are also significantly (p < 0.05) correlated (Table S5) in cluster-2 (Fig. S3a). Industrial (e.g., coal-burning in brickfields, leather tanning, natural oil-gas reserving, textile dying, etc.), urban (e.g., waste disposals, sewerage leakage, etc.) as well as agricultural activities (e.g., administration of fertilizers and pesticides) can reasonably be assumed to enrich the level of Cr, Co, and Cd in groundwater [5,6,49,108], from where the tap water has been supplied to the schools and colleges.

On the other hand, components PC3, PC4, and PC5, cumulatively represent 33.53% of the total variance which can explain the several aspects of trace metals from supply pipe scales and loose deposits [109]. PC3, accounted for 11.78% of the total variance, with the strong loading for Mn and moderate loading of Fe and Zn indicating that Fe–Mn-oxides of pipe scale play a significant role in the accumulation and release of Zn. Similarly, PC4 represents 10.91% of the total variance which demonstrated strong positive loadings of Fe and Pb which is also indicating the Fe-oxides-based accumulation followed by releasing of Pb into the tap water. However, PC5 can explain 10.85% of the variance where Ni and As are strongly loaded which are released into the tap water from the galvanized materials of supply pipelines. Metal(loid)s that are strongly and/or moderately loaded in PC3-5 are also belonged to cluster-3 (Fig. S3a). However, trace metal(loid)s in cluster-3 are almost insignificantly correlated (Table S5) with each other. Hence, these metal(loid)s were assumed to be released from a single source (supply pipelines) having diverse independent chemical mechanisms of trace metal accumulation and release. However, as the pipe scaling is directly correlated to the age of the pipeline (considering the identical pH range over the considered period), older schools and colleges are supposed to have higher trace metals in tap-water. In the hierarchical cluster analysis (CA) for the sampling sites (Fig. S3a), two significantly separated clusters are identified, where cluster-1 and cluster-2 are representing relatively older and newly established schools and colleges, respectively (Table S1). On average, schools and colleges belonging to cluster-1 were established in ∼1980 whereas schools and colleges belonging to cluster-2 were established in ∼2000. Average abundances of trace metal(loid)s in tap-water of cluster-1 schools and colleges (Cr: 3.34 μgL^−1^; Mn: 37 μgL^−1^; Fe: 192 μgL^−1^; Co: 0.72 μgL^−1^; Ni: 12.0 μgL^−1^; Zn: 82.8 μgL^−1^; As: 1.92 μgL^−1^; Cd: 0.063 μgL^−1^; Pb: 6.23 μgL^−1^) are relatively higher compared to those of schools in cluster-2 (Cr: 2.65 μgL^−1^; Mn: 44 μgL^−1^; Fe: 139 μgL^−1^; Co: 0.50 μgL^−1^; Ni: 10.8 μgL^−1^; Zn: 149.6 μgL^−1^; As: 0.57 μgL^−1^; Cd: 0.052 μgL^−1^; Pb: 5.81 μgL^−1^).

### 3.5 Health risk assessment

**Fig. 3:**
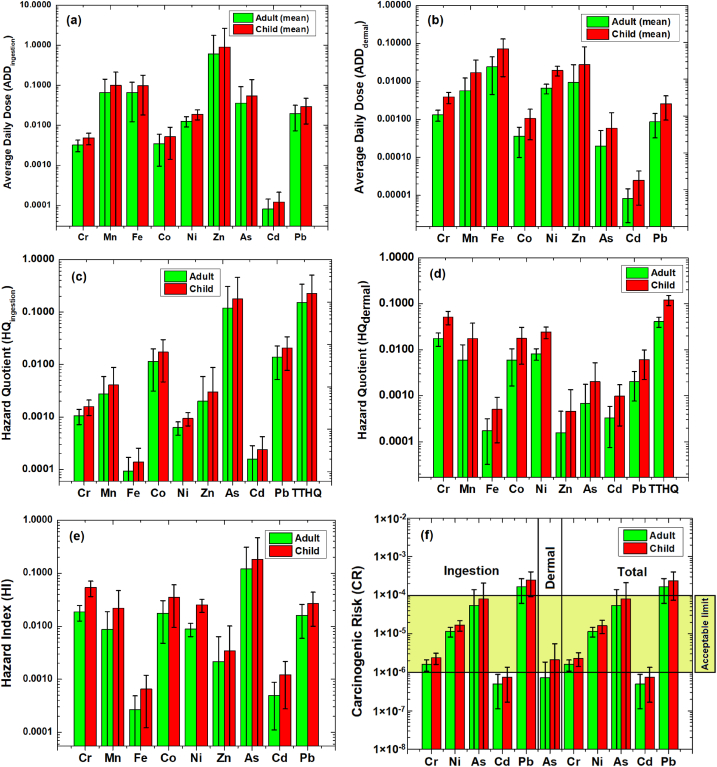
Non-carcinogenic and carcinogenic health risks appraisals for the dissolved trace metal(oid)s in tap water collected from educational institutions of the study area.

Although in most of the cases, abundances of metal(loid)s in the studied tap-water were within the limits of national and international recommendations, this study has estimated the health risks to understand their levels and to intend some potential remediation approaches. In doing so, both the ingestion and dermal exposures are considered for estimating the non-carcinogenic (NCR) and carcinogenic health risks (CR) originated from the studied trace metal(loid)s. Average daily doses for ingestion (ADD_ingestion_) and dermal (ADD_dermal_) exposure for adults range from 7.99 × 10^−5^ (Cd) to 6.00 × 10^−1^ (Zn) and 8.34 × 10^−6^ (Cd) to 2.44 × 10^−2^ (Fe) mg kg^−1^ day^−1^, respectively (Fig. 3a). However, ADD_ingestion_ and ADD_dermal_ for children range from 1.19 × 10^−4^ (Cd) to 8.96 × 10^−1^ (Zn) and 2.46 × 10^−5^ (Cd) to 7.21 × 10^−2^ (Fe) mg kg^−1^ day^−1^, respectively (Fig. 3b). ADD for ingestion are 1.9 (Ni)–182 (As) and 1.0 (Ni)–92.1 (As) times higher compared to the corresponding dermal exposure for adults and children, respectively. ADD_ingestion_ and ADD_dermal_ are mostly governed by the gastrointestinal absorption factor (%) and the dermal permeability coefficient (cm h^−1^), respectively. ADD-values for both-way exposures are higher for the child compared to the adult.

Hazard quotients (HQ_ingestion_ and HQ_dermal_) and total target hazard quotients (TTHQ_ingestion_ and TTHQ_dermal_) for both-way exposures are less than one (Fig. 3c and d) and these estimated parameters possess relatively higher values for children compared to the adults. Consequently, the total non-carcinogenic health risks represented by mean hazard index (HI; Adult: 1.57 × 10^−4^ – 1.75 × 10^−2^; Child: 4.62 × 10^−4^ – 5.17 × 10^−2^) estimated for the studied tap water samples are lower than the threshold value [64] (Fig. 3e). However, the hazard index (HI= HQ_ingestion_ + HQ_dermal_) for older schools (established ∼1980, cluster-1) (HI_Adult_: 0.24; HI_Child_: 0.43) are ∼2 times higher compared to those of newly established schools (established ∼2000, cluster-2) (HI_Adult_: 0.12; HI_Child_: 0.23) (Table S1; Fig. S3b) [[110], [111], [112], [113]]. Thus, considering this progressive deterioration, identical water abstraction, and supply processes, the load of the trace metal(loid)s dissolved in the tap-water can cause potential non-carcinogenic health risks to the students in the future.

On the other hand, dissolved Pb in the analyzed tap water represented carcinogenic risks (CR_Pb-Adult_: 1.65 × 10^−4^; CR_Pb-Child_: 2.37 × 10^−4^) while considering the upper limit of the estimated uncertainty (standard deviations, n = 25), As can also cause cancer (CR_As-Adult_: 5.44 × 10^−5^; CR_As-Child_: 8.06 × 10^−5^) (Fig. 3f). Nevertheless, both NCR and CR depend on body weight, water consumption, and exposure durations, which can introduce uncertainty in our risk estimation. Thus, in both cases (NCR and CR) and through the differential exposure pathways, currently, the supplied tap water of the studied educational institutions may cause trivial health risks, although the younger age group is more vulnerable compared to the adults. However, proper precaution approaches (e.g., changing the supply pipelines every 1-2 decades, using a water purifier to obtain potable water, etc.) should be adopted in the future to alleviate the water-borne health risks.

## 4 Summary and limitations

This work, for the first time, reveals the abundances of the metal(loid)s dissolved in the tap-water samples collected from different educational institutions located in a highly dense demographic area of Bangladesh (Mirpur, Dhaka). Measured elemental abundances in tap water were mostly within the local and international threshold limits which in turn provide ‘excellent’ quality of potable water in terms of studied metal(loid)s, with seldom exceptions. However, dissolved metals' (Na, Mg, Ni, Zn, and Pb) loads were higher compared to those of relevant literature data across the globe. Multivariate statistical approaches indicated that major elemental abundances were sourced from geogenic incorporations, e.g., dolomite weathering and/or corresponding water-rock interactions. However, PCA and cluster analysis on the elemental abundances identified anthropogenic sources of Cr, Co, and Cd, which were also significantly correlated among themselves. On the other hand, dissolved Ni, As, Zn, Mn, Fe, and Pb in the examined tap water were originated from the pipeline scaling, where varying independent mechanisms of metal(loid)s’ adsorption followed by releasing were invoked. The occurrence of the independent mechanisms of metal(loid)s’ exchange between pipe-scale and water was also supported by the loadings of Ni, As, Zn, Mn, Fe, and Pb in different components of PCA as well as their insignificant correlations. Furthermore, cluster analysis on the sampling sites differentiated two groups of schools where older schools possess relatively higher levels of metal(loid)s. Considering the pipeline scaling is a linear function of time duration, cluster analysis reasonably identified such events in metal(loid)s'incorporation to the tap water. Preliminary health risks appraisals demonstrated that non-carcinogenic risks are unlikely to occur due to the metal(loid)s dissolved in tap-water whereas Pb and A can cause carcinogenic health risks on continuous exposures. In terms of both carcinogenic and non-carcinogenic health risks, younger age group is more vulnerable toward the dissolved metal(loid)s in tap-water compared to the adults. Although the health risks estimation demonstrated trivial risks, preventive steps should be taken for future deterioration of water quality.

This study invokes some future study on a temporal scale for further ensuring the pipe-line-scaling governing metal(loid)s’ transfer. Along with the continuous monitoring, specific studies on supply water pipe-line and the independent mechanism of metal(loid)s' adsorption followed by transfer should be investigated.

## Funding

No external funding received.

## Author contribution statement

Md. Joynal Abedin: Performed the experiments, Wrote the paper.

Rahat Khan: Conceived and designed the experiments, Analyzed and interpreted the data, Wrote the paper.

Md. Abu Bakar Siddique: Analyzed and interpreted the data, Contributed reagents, materials, analysis tools or data.

Abdul Hadi Al Nafi Khan, Md. Tariqul Islam, Md. Bazlar Rashid: Contributed analysis tools or data, interpreted the data, Wrote the paper.

## Data availability statement

Data included in article/supplementary material/referenced in article.

## Declaration of interest's statement

The authors declare that they have no known competing financial interests or personal relationships that could have appeared to influence the work reported in this paper.
