# Supplementary material for: Metal(loid)s in tap-water from schools in central Bangladesh (Mirpur): Source apportionment, water quality, and health risks appraisals
Source: Heliyon. 2023 Apr 28;9(5):e15747. doi: 10.1016/j.heliyon.2023.e15747 (PMC10189184; doi:10.1016/j.heliyon.2023.e15747)
Supplement: Multimedia component 1 [file mmc1.docx]

**Table S1** Samples information of the studied area (Mirpur, Bangladesh) along with their ancillary data.

| Sample ID |  | School Name |  | Year of Establishment |  | GPS data | |
| --- | --- | --- | --- | --- | --- | --- | --- |
|  |  |  |  |  |  | Latitude | Longitude |
| S-1 |  | Monipur High School & College (Br-2) |  | 1969 |  | 23°47'49.5"N | 90°23'02.9"E |
| S-2 |  | Shaheed Police Srmiti School and College |  | 1999 |  | 23°47'59.6"N | 90°23'05.0"E |
| S-3 |  | Rotary School and College |  | 1976 |  | 23°48'01.5"N | 90°22'51.3"E |
| S-4 |  | Glory School and College-1 |  | 2006 |  | 23°47'04.2"N | 90°22'28.6"E |
| S-5 |  | Banaful Adibasi Green Heart College |  | 1976 |  | 23°48'19.7"N | 90°22'33.8"E |
| S-6 |  | Mirpur Girls Ideal College |  | 1978 |  | 23°48'26.5"N | 90°22'17.0"E |
| S-7 |  | Mirpur Adarsho High School |  | 1966 |  | 23°48'23.0"N | 90°22'14.8"E |
| S-8 |  | Senpara Porbota Govt Primary School |  | 1943 |  | 23°48'27.9"N | 90°22'13.0"E |
| S-9 |  | Monipur High School and College (Girls) |  | 1969 |  | 23°48'00.8"N | 90°21'49.1"E |
| S-10 |  | Monipur Govt Primary School |  | 1966 |  | 23°48'00.9"N | 90°21'55.6"E |
| S-11 |  | Sheikh Fazilatunnesa Mohila College |  | 1980 |  | 23°48'16.2"N | 90°21'35.1"E |
| S-12 |  | Mirpur College |  | 1970 |  | 23°48'14.9"N | 90°21'39.1"E |
| S-13 |  | National Bangla High School |  | 1966 |  | 23°48'18.5"N | 90°21'44.1"E |
| S-14 |  | Paikpara Govt Primary School |  | 1986 |  | 23°47'36.8"N | 90°21'29.9"E |
| S-15 |  | Wak-Up High School |  | 2000 |  | 23°47'23.6"N | 90°21'11.3"E |
| S-16 |  | Wak Up Govt Primary School |  | 1989 |  | 23°47'24.6"N | 90°21'10.2"E |
| S-17 |  | Islamia High School |  | 1995 |  | 23°48'45.5"N | 90°21'33.8"E |
| S-18 |  | Candour International School |  | 1999 |  | 23°49'30.5"N | 90°21'54.3"E |
| S-19 |  | Mirpur Cantonment Public School and College |  | 2014 |  | 23°50'06.2"N | 90°21'53.5"E |
| S-20 |  | SOS Hermann Gmeiner School and College |  | 1986 |  | 23°48'17.7"N | 90°22'38.6"E |
| S-21 |  | Cosmo School |  | 2013 |  | 23°49'21.3"N | 90°21'52.9"E |
| S-22 |  | Heed International School |  | 2003 |  | 23°49'03.3"N | 90°21'57.9"E |
| S-23 |  | Shaheed Abu Taleb High School |  | 1975 |  | 23°48'35.8"N | 90°22'09.8"E |
| S-24 |  | Glory School and College-2 |  | 2006 |  | 23°48'10.6"N | 90°22'42.0"E |
| S-25 |  | Mirpur English version School and College |  | 2013 |  | 23°48'24.6"N | 90°22'22.8"E |

**Table S2** Experimental parameters of the element-specific hollow cathode lamp used in the AAS instrument during the measurements of the samples along with the limit of quantification (LOQ), the limit of detection (LOD), calibration range, and measurement uncertainty for all the analysed chemical elements.

| Elements | Experimental parameters of hollow cathode lamp | | | LOQ  (µg/L) | LOD  (µg/L) | Calibration range (µg/L) | Measurement uncertainty (±) (K=2) |
| --- | --- | --- | --- | --- | --- | --- | --- |
|  | Lamp current (mA) | Wavelength (nm) | Slit width (nm) |  |  |  |  |
| Fe | 5.0 | 248.3 | 0.2 | 100 | 67.7 | 100-2000 | 8% |
| Mn | 5.0 | 279.5 | 0.2 | 50 | 3.1 | 50-1000 | 7% |
| Zn | 5.0 | 213.9 | 1.0 | 50 | 7.5 | 50-1000 | 11.6% |
| Ca | 10.0 | 422.7 | 0.5 | 100 | 24.1 | 100-2000 | 8.4% |
| Mg | 4.0 | 285.2 | 0.5 | 100 | 6.3 | 100-400 | 5.2% |
| Na | - | - | - | 200 | 373.8 | 200-4000 | 11.4% |
| K | - | - | - | 200 | 168.7 | 200-4000 | 21.6% |
| Co | 7.0 | 240.7 | 0.2 | 25 | 2.1 | 25-75 | 21% |
| Pb | 10.0 | 217.0 | 1.0 | 5 | 1.2 | 5-15 | 31% |
| As | 10.0 | 193.7 | 0.5 | 2 | 0.2 | 2-20 | 16% |
| Ni | 4.0 | 232.0 | 0.2 | 25 | 2.2 | 25-75 | 23.4% |
| Cd | 4.0 | 228.8 | 0.5 | 1 | 0.3 | 1-3 | 16.8% |
| Cr | 7.0 | 357.9 | 0.2 | 5 | 0.4 | 5-15 | 20.8% |

N.B.: Na and K were analyzed in natural gas flame in a Flame Photometer without using any hollow cathode lamp.

**Table S3** Health risk assessment factors including reference values.

|  |  |  | Unit | Adults |  | Children |  | References | |
| --- | --- | --- | --- | --- | --- | --- | --- | --- | --- |
| Ingestion rate (IR) | | | L/day | 2 |  | 0.64 |  | [48, 59, 103, 104] |  |
| Exposure frequency (EF) | | | days/year | 350 |  | 350 |  | [48, 59, 104] |  |
| Exposed skin area (SA) | | | cm^2^ | 18000 |  | 6600 |  | [48, 59, 104] |  |
| Exposure time (ET) | | | h/day | 0.58 |  | 1 |  | [48, 59, 103] |  |
| Exposure duration (ED) | | | years | 70 |  | 6 |  | [64, 104] |  |
| Body weight (BW) | | | kg | 70 |  | 15 |  | [48, 59, 105] | |
| Average time for non-carcinogens (AT) | | | days | 25550 |  | 2190 |  | [64, 104] |  |

| Element | Gastrointestinal absorption factor (Abs_g_ in %) | References | Dermal permeability  coefficient (K_p_ in cm/h) | References |
| --- | --- | --- | --- | --- |
| Cr | 3.8 | [103, 104] | 3.00×10^-3^ | [64] |
| Mn | 6 | [103, 104] | 1.00×10^-3^ | [64] |
| Fe | 1.4 | [103, 104] | 1.00×10^-3^ | [64] |
| Co | 20 | [106] | 4.00×10^-3^ | [63] |
| Ni | 4 | [103, 104] | 4.00×10^-3^ | [63] |
| Cu | 57 | [103, 104] | 1.00×10^-3^ | [64] |
| Zn | 20 | [103, 104] | 6.00×10^-4^ | [64] |
| As | 95 | [103, 104] | 1.00×10^-3^ | [64] |
| Cd | 5 | [103, 104] | 1.00×10^-3^ | [64] |
| Hg | 7 | [64] | 1.00×10^-3^ | [64] |
| Pb | 11.7 | [103, 104] | 1.00×10^-3^ | [63] |

|  | R_f_D_ingestion_ | R_f_D_dermal_ | References | |  |  | Oral cancer slope factor (SF) (µg/kg/day)^-1^ | |  | References |
| --- | --- | --- | --- | --- | --- | --- | --- | --- | --- | --- |
|  | (µg kg^-1^ day^-1^) | (µg kg^-1^ day^-1^) |  |  |  |  | Ingestion | Dermal |  |  |
| Cr | 3 | 0.075 |  |  |  |  | 0.0005 |  |  | [65] |
| Mn | 24 | 0.96 |  |  |  |  |  |  |  |  |
| Fe | 700 | 140 |  |  |  |  |  |  |  |  |
| Co | 0.3 | 0.06 |  |  |  |  |  |  |  |  |
| Ni | 20 | 0.8 | [103] | | | | 0.00091 |  |  | [63] |
| Cu | 40 | 12 |  |  |  |  |  |  |  |  |
| Zn | 300 | 60 |  |  |  |  |  |  |  |  |
| As | 0.3 | 0.285 |  |  |  |  | 0.0015 | 0.00366 |  | [66, 67] |
| Cd | 0.5 | 0.025 |  |  |  |  | 0.0063 |  |  | [105, 65] |
| Hg | 0.3 | 0.021 | [66, 67] | | | |  |  |  |  |
| Pb | 1.4 | 0.42 | [104] | | | | 0.0085 |  |  | [66, 67] |

**Table S4** Varimax rotated factor loadings and communalities of the analyzed chemical elements (strong loadings are in bold face).

| **Parameters** | PC1 | PC2 | PC3 | PC4 | PC5 | Communalities |
| --- | --- | --- | --- | --- | --- | --- |
| Na | **0.960** | 0.015 | -0.125 | 0.005 | 0.094 | 0.946 |
| Mg | **0.953** | -0.046 | -0.117 | -0.056 | 0.097 | 0.936 |
| K | **0.757** | -0.108 | 0.529 | 0.090 | -0.049 | 0.875 |
| Ca | **0.938** | -0.115 | -0.123 | -0.107 | -0.074 | 0.926 |
| Cr | 0.165 | **0.773** | -0.233 | -0.099 | 0.317 | 0.79 |
| Mn | -0.127 | -0.155 | **0.796** | 0.059 | -0.043 | 0.679 |
| Fe | 0.032 | 0.050 | 0.434 | **0.779** | 0.087 | 0.807 |
| Co | -0.120 | **0.825** | -0.017 | 0.326 | -0.106 | 0.812 |
| Ni | -0.202 | -0.128 | -0.101 | -0.164 | **0.818** | 0.764 |
| Zn | -0.234 | 0.335 | 0.321 | -0.068 | 0.099 | 0.285 |
| As | 0.360 | 0.158 | 0.151 | 0.147 | **0.723** | 0.723 |
| Cd | -0.116 | **0.775** | 0.004 | -0.228 | -0.141 | 0.686 |
| Pb | -0.159 | -0.173 | -0.441 | **0.751** | -0.203 | 0.854 |
| Eigenvalue | 3.673 | 2.168 | 1.601 | 1.458 | 1.183 |  |
| % of total variance | 27.740 | 16.280 | 11.779 | 10.907 | 10.847 |  |
| Cumulative % of variance | 27.740 | 44.021 | 55.800 | 66.707 | 77.554 |  |

**Table S5** Pearson correlation matrix for the analyzed metal(loid)s.

| Parameters | Na | Mg | K | Ca | Cr | Mn | Fe | Co | Ni | Zn | As | Cd | Pb |
| --- | --- | --- | --- | --- | --- | --- | --- | --- | --- | --- | --- | --- | --- |
| Na | 1 |  |  |  |  |  |  |  |  |  |  |  |  |
| Mg | 0.952^**^ | 1 |  |  |  |  |  |  |  |  |  |  |  |
| K | 0.623^**^ | 0.635^**^ | 1 |  |  |  |  |  |  |  |  |  |  |
| Ca | 0.890^**^ | 0.910^**^ | 0.637^**^ | 1 |  |  |  |  |  |  |  |  |  |
| Cr | 0.193 | 0.184 | -0.124 | 0.063 | 1 |  |  |  |  |  |  |  |  |
| Mn | -0.190 | -0.189 | 0.250 | -0.192 | -0.232 | 1 |  |  |  |  |  |  |  |
| Fe | -0.041 | -0.036 | 0.323 | -0.107 | -0.086 | 0.280 | 1 |  |  |  |  |  |  |
| Co | -0.064 | -0.178 | -0.179 | -0.238 | 0.504^*^ | -0.050 | 0.195 | 1 |  |  |  |  |  |
| Ni | -0.091 | -0.028 | -0.198 | -0.165 | 0.131 | -0.038 | -0.039 | -0.189 | 1 |  |  |  |  |
| Zn | -0.237 | -0.217 | 0.001 | -0.248 | 0.021 | -0.043 | 0.033 | 0.231 | 0.021 | 1 |  |  |  |
| As | 0.394 | 0.303 | 0.273 | 0.207 | 0.313 | 0.018 | 0.132 | 0.045 | 0.244 | 0.094 | 1 |  |  |
| Cd | -0.125 | -0.133 | -0.148 | -0.133 | 0.490^*^ | -0.079 | -0.068 | 0.469^*^ | -0.021 | 0.162 | -0.096 | 1 |  |
| Pb | -0.107 | -0.136 | -0.234 | -0.114 | -0.211 | -0.174 | 0.270 | 0.109 | -0.154 | -0.128 | -0.170 | -0.183 | 1 |
| **. Correlation is significant at the 0.01 level (2-tailed).  *. Correlation is significant at the 0.05 level (2-tailed). | | | | | | | | | | | |  |  |


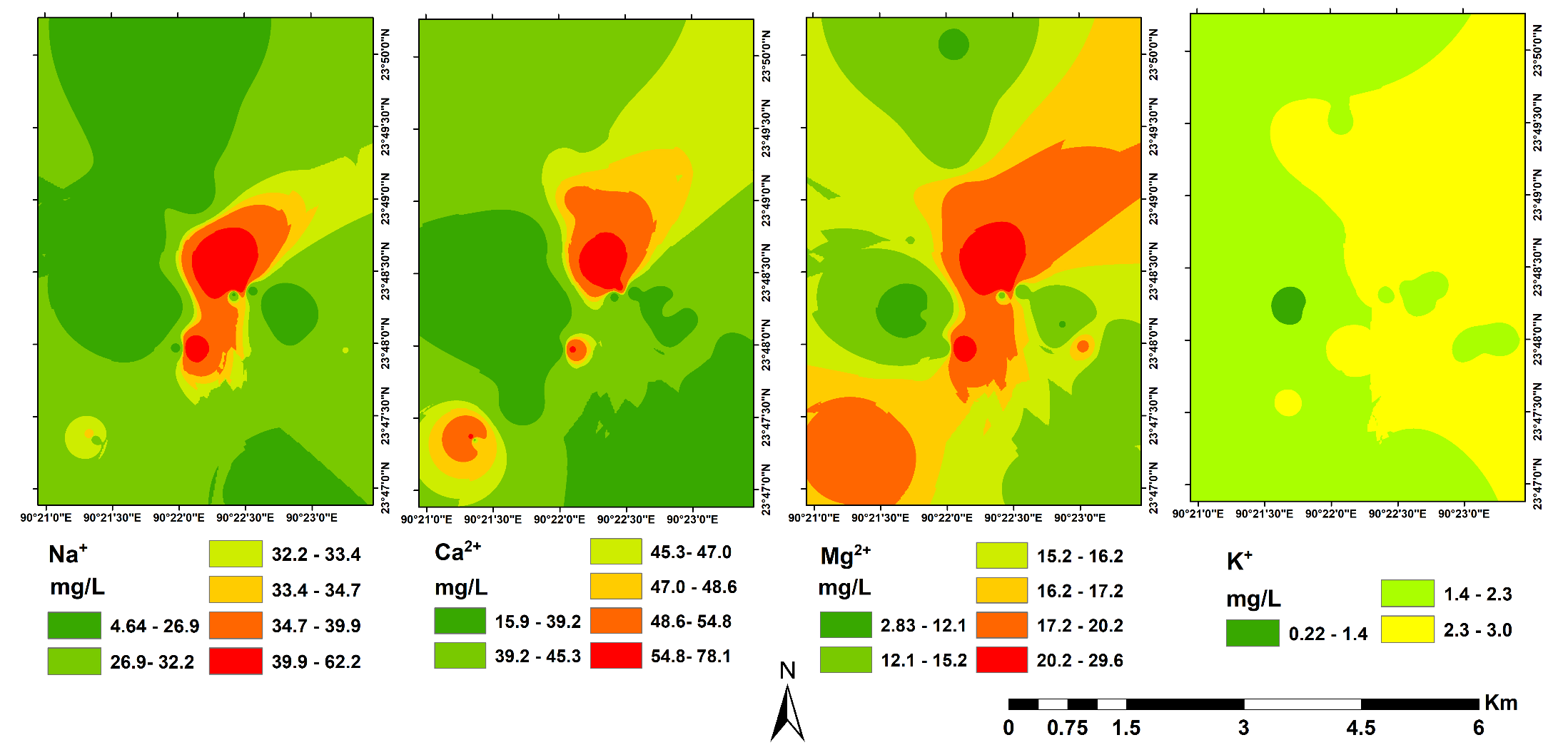


**Fig. S1** Major elemental distributions in the collected tap water samples.


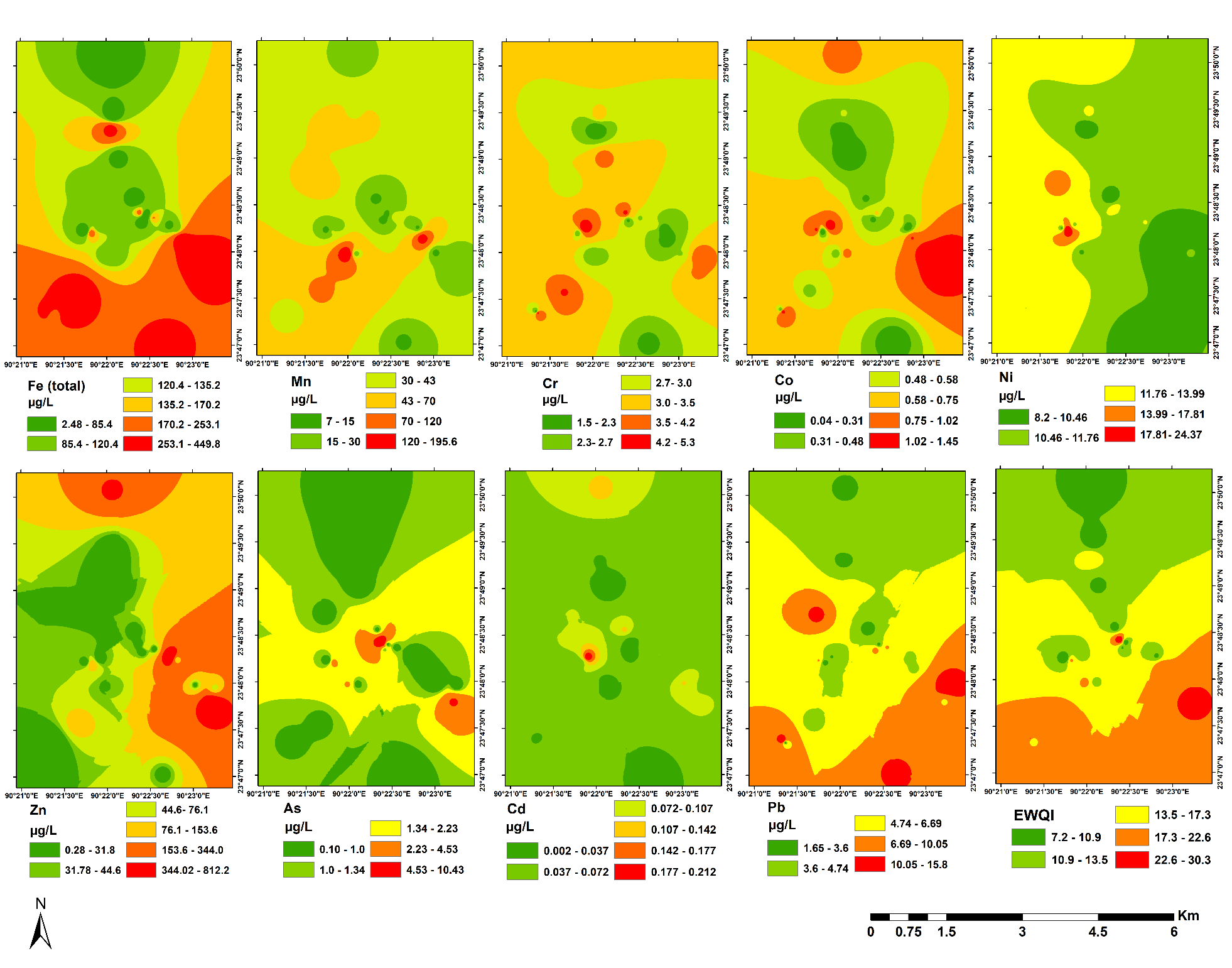


**Fig. S2** Trace elemental distributions in the collected tap water samples.


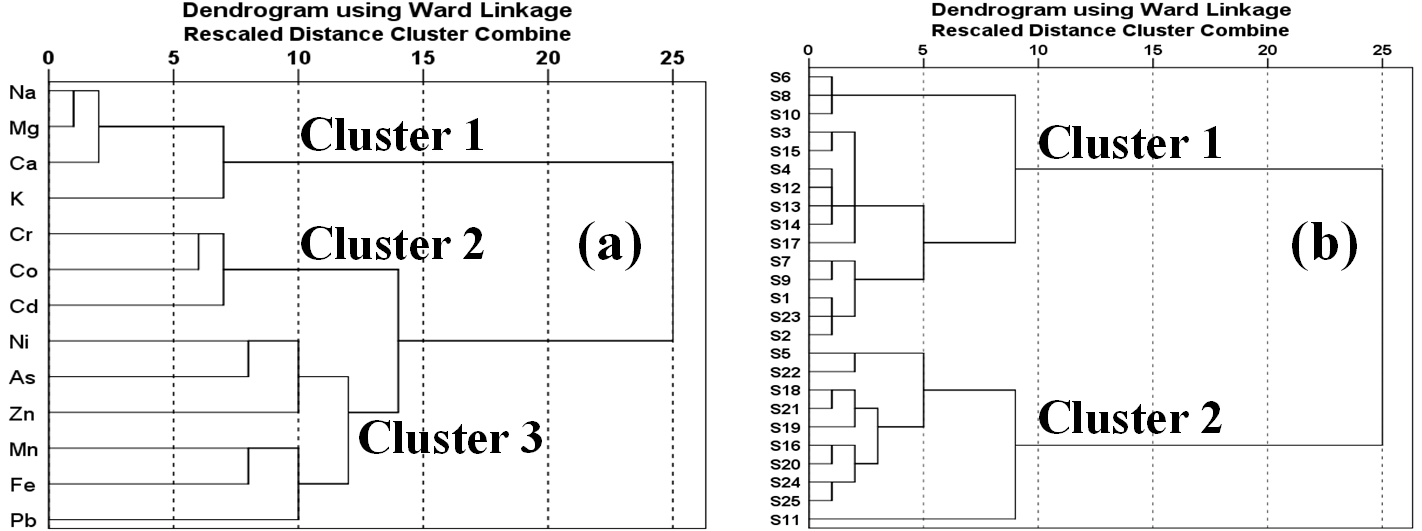


**Fig. S3** Dendrogram obtained by hierarchical clustering analysis for (a) the analyzed metal(loid)s and (b) the sampling sites.
